# Continuous Flow Atmospheric Pressure Laser Desorption/Ionization Using a 6–7-µm-Band Mid-Infrared Tunable Laser for Biomolecular Mass Spectrometry

**DOI:** 10.3390/ijms150610821

**Published:** 2014-06-16

**Authors:** Ryuji Hiraguchi, Hisanao Hazama, Kenichirou Senoo, Yukinori Yahata, Katsuyoshi Masuda, Kunio Awazu

**Affiliations:** 1 Graduate School of Engineering, Osaka University, 2-1 Yamadaoka, Suita, Osaka 565-0871, Japan; E-Mails: hiraguchi-r@mb.see.eng.osaka-u.ac.jp (R.H.); awazu@see.eng.osaka-u.ac.jp (K.A.); 2 JEOL Ltd., 1156 Nakagamicho, Akishima, Tokyo 196-0022, Japan; E-Mails: ksenoo@jeol.co.jp (K.S.); yyahata@jeol.co.jp (Y.Y.); 3 Suntory Institute for Bioorganic Research, Suntory Foundation for Life Sciences, 1-1-1 Wakayamadai, Shimamotocho, Mishimagun, Osaka 618-0024, Japan; E-Mail: katsuyoshimasuda@hotmail.co.jp; 4 Graduate School of Frontier Biosciences, Osaka University, 1-1 Yamadaoka, Suita, Osaka 565-0871, Japan; 5 The Center of Advanced Medical Engineering and Informatics, Osaka University, 2-2 Yamadaoka, Suita, Osaka 565-0871, Japan

**Keywords:** mass spectrometry, atmospheric pressure laser ionization, mid-infrared tunable laser, peptides

## Abstract

A continuous flow atmospheric pressure laser desorption/ionization technique using a porous stainless steel probe and a 6–7-µm-band mid-infrared tunable laser was developed. This ion source is capable of direct ionization from a continuous flow with a high temporal stability. The 6–7-µm wavelength region corresponds to the characteristic absorption bands of various molecular vibration modes, including O–H, C=O, CH_3_ and C–N bonds. Consequently, many organic compounds and solvents, including water, have characteristic absorption peaks in this region. This ion source requires no additional matrix, and utilizes water or acetonitrile as the solvent matrix at several absorption peak wavelengths (6.05 and 7.27 µm, respectively). The distribution of multiply-charged peptide ions is extremely sensitive to the temperature of the heated capillary, which is the inlet of the mass spectrometer. This ionization technique has potential for the interface of liquid chromatography/mass spectrometry (LC/MS).

## 1. Introduction

Currently, electrospray ionization (ESI) [1,2] and matrix-assisted laser desorption/ionization (MALDI) [3,4] are the two major ionization methods for large biomolecules. Although these methods are used in combination with other separation techniques (e.g., liquid chromatography) for proteome analysis, they have some limitations. ESI can continuously ionize flowing liquid samples, but ionization is unstable for low volatility solvents, such as buffer solutions. On the other hand, conventional MALDI is limited to dry samples, because the MALDI process is conducted under a high vacuum, and co-crystallization of the analyte and aromatic matrix leads to spatial inhomogeneity and a low reproducibility. In fact, conventional soft ionization methods for biological polymers cannot produce a highly stable ionization with low volatility solvents. Consequently, novel ionization techniques are necessary for high-throughput and reproducible proteome analysis.

To overcome the above issues, the solvent, the state in conventional ionization methods (ESI and MALDI) and various original ambient ionization methods that can simplify or omit the sample preparation process have been intensively investigated. A few methods have been reported at atmospheric pressure. Ionization under atmospheric pressure can tolerate various sample states, because the samples do not have to be dry. Additionally, electrospray laser desorption ionization (ELDI) [5] generates gas phase ions when an untreated tissue sample is irradiated with an ultraviolet (UV) laser under atmospheric pressure (AP), and the charged liquid droplets by the electrospray act to desorbed neutral molecules and ions. Unlike ELDI, matrix-assisted laser desorption electrospray ionization (MALDESI) [6] increases the ionization efficiency by adding an aromatic matrix. Laser ablation electrospray ionization (LAESI) [7] enables lipids from untreated biological samples to be directly ionized using the intensive absorption of an infrared (IR) laser by a tissue and electrospray, while modified ESI techniques without laser ablation/desorption have been developed for surface analysis. Desorption electrospray ionization (DESI), which was initially reported by Cooks *et al.*, enables proteins, peptides, *etc*., to be directly analyzed from the surfaces of tissues and plants under ambient conditions [8]. DESI can also be used with thin-layer chromatography [9]. Probe electrospray ionization (PESI), which was developed by Hiraoka *et al.*, has a sampling system where a solid needle moves along a vertical axis by a motor-driven system [10]. PESI has the potential to analyze proteins directly from salt/urea-contaminated solutions [11].

However, these ambient ionization methods using laser desorption cannot ionize a liquid state sample directly. To realize high-throughput proteomics with a high reproducibility, an ambient laser ionization method for liquid state samples with low volatility solvents is desired.

Although conventional MALDI has been performed using UV lasers, IR-MALDI [12,13,14] has been investigated, because it may utilize more diverse matrices; many organic compounds with strong characteristic absorption peaks in the mid-IR wavelength range have been proposed as new matrices.

On the other hand, attempts to conduct the MALDI process under atmospheric pressure have been executed in order to expand the range of the analytical protocol. Laiko *et al.* presented an AP-MALDI technique coupled with an orthogonal acceleration time-of-flight (oaTOF) mass spectrometer and an ion trap mass spectrometer [15,16,17]. AP-MALDI has a better softness than conventional vacuum MALDI due to the collisional cooling of the expanding plume. In addition, alternating the surrounding condition to atmospheric pressure may expand AP-MALDI to include samples containing volatile compounds. In fact, online analysis of a solution sample by AP-MALDI has been demonstrated [18,19].

One of the most promising IR matrices is water, because it is abundant, environmentally benign and has a strong absorption in the mid-IR region. In 2002, Laiko *et al.* demonstrated the atmospheric pressure ionization of peptides in an aqueous solution using a Yb:YAG laser-pumped optical parametric oscillator (OPO) with a fixed wavelength of 3 µm [20]. Their work strongly suggests that utilizing a solvent as a light-absorbing matrix can expand the target samples in atmospheric pressure infrared matrix-assisted laser desorption ionization (AP-IR-MALDI) to include natural (aqueous) solutions. AP-IR-MALDI using a mid-IR OPO with a 2.9-µm wavelength has been applied to online liquid chromatography/mass spectrometry (LC/MS) analysis of the tryptic digest of bovine serum albumin [21]. Several works have employed 3-µm-band lasers, including OPO pumped by a Nd:YAG laser and an Er:YAG laser with a 2.94-µm wavelength for AP-IR-MALDI. This wavelength corresponds to the absorption peak of the O–H stretching vibration of liquid water.

The mid-IR wavelength region corresponds to the characteristic absorption bands of various molecular vibration modes, including O–H, C=O, CH_3_ and C–N bonds. Consequently, many organic compounds and solvents, including water, have characteristic absorption peaks in this region (e.g., acetonitrile, methanol, acetone and several buffer solutions, such as a phosphate buffer, acetic acid buffer and Tris buffer). For example, the O–H bending vibration for liquid water has an absorption peak at a wavelength of 6.07 µm. Therefore, 6–7-µm-band mid-IR lasers should be able to utilize diverse solvents as a matrix compared to 3-µm-band lasers.

In fact, a 6-µm-band mid-infrared tunable laser using difference-frequency generation (DFG), which utilizes energy absorption in the C=O stretching region, has been applied to vacuum MALDI [22], and the softness for labile molecules (e.g., polysulfated oligosaccharides or polysialylated gangliosides) appears to exceed those of all other UV MALDI methods [23]. Simultaneous irradiations of a UV laser and 6-µm-band mid-IR free electron laser (FEL) enable protein samples containing a denaturant at a high concentration to be analyzed [24]. Awazu *et al.* have demonstrated a promising technique of IR-MALDI using a DFG laser utilizing various compounds (e.g., urea) as a matrix [25]. Although a 6–7-µm-band mid-infrared laser may utilize organic compounds, as well as various other solvents as a matrix when the MALDI process is conducted at atmospheric pressure, an AP-IR-MALDI method using a 6–7-µm-band infrared laser has yet to be reported.

In this study, we developed a new atmospheric pressure laser ionization method using a novel 6–7-µm-band mid-IR tunable laser. Our method allows the mass spectra of peptides to be directly measured from the continuous flow of several solutions.

## 2. Results and Discussion

### 2.1. Temporal Stability of Continuous Flow (CF) Ionization of Peptides

**Figure 1:**
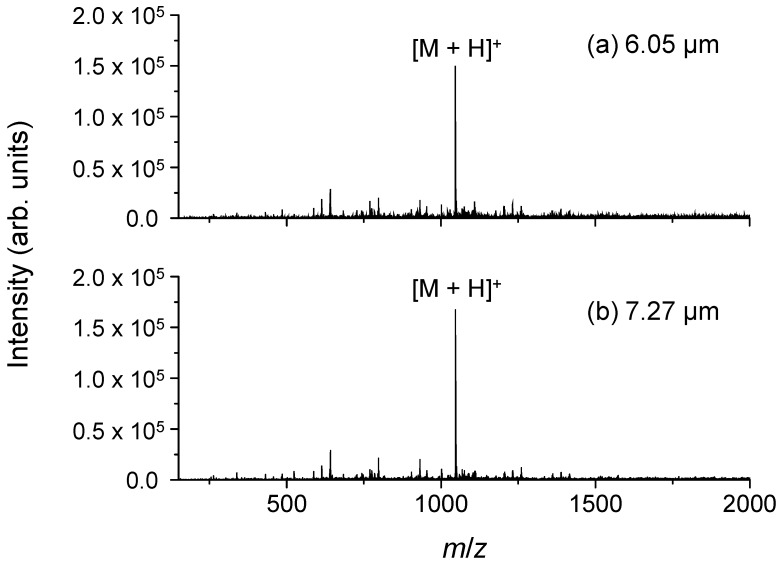
Typical mass spectra from angiotensin II dissolved in an 80% acetonitrile aqueous solution upon irradiation with a mid-infrared laser at wavelengths of (**a**) 6.05 µm and (**b**) 7.27 µm. The electric potential of the frit and the capillary temperature were set to 2.5 kV and 270 °C, respectively.

**Figure 2:**
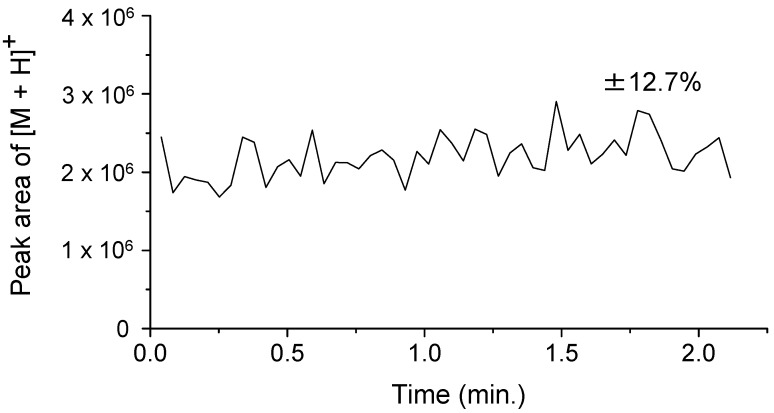
Extracted ion chromatogram of the protonated ion [M + H]^+^ of angiotensin II. The wavelength of the DFG laser is set at 6.05 µm, and the pulse energy is 400 µJ. Each data point was extracted from the raw mass spectra, which were averaged from three micro-scans. The electric potential of the frit and the capillary temperature were set to 2.5 kV and 270 °C, respectively.

We initially examined the adequacy of the temporal stability of the ion signal intensity. Figure 1 shows a typical result of continuous ionization with a 5-µL/min sample flow and laser irradiation at 6.05 or 7.27 µm. The singly protonated ion [M + H]^+^ of angiotensin II is predominantly observed. In addition, the extracted ion chromatogram of [M + H]^+^ obtained from 50 mass spectra shows a superior stability of the ionization process compared with conventional MALDI using a solid matrix (Figure 2). The variability of the ion signal intensity has a standard deviation of 12.7%. On the other hand, the DFG laser has a variation of up to 10% in the output energy. In addition, the temporal relationship between laser pulses and injection time of the ion trap may be responsible for these variations, because the lasers were operated without synchronization. Thus, the sequential ion signal from the solution at a surface near the frit is responsible for the continuous flow ionization. This result suggests that this technique may be a novel interface in LC and MS. On the other hand, the efficiency of sample desorption depends strongly on the parameters of the laser and solvent. Thus, we then investigated the relationships between the laser wavelength, pulse energy, absorption coefficient of the solvent and ionization efficiency.

### 2.2. Wavelength Dependence of the Ion Signal Intensity

**Figure 3:**
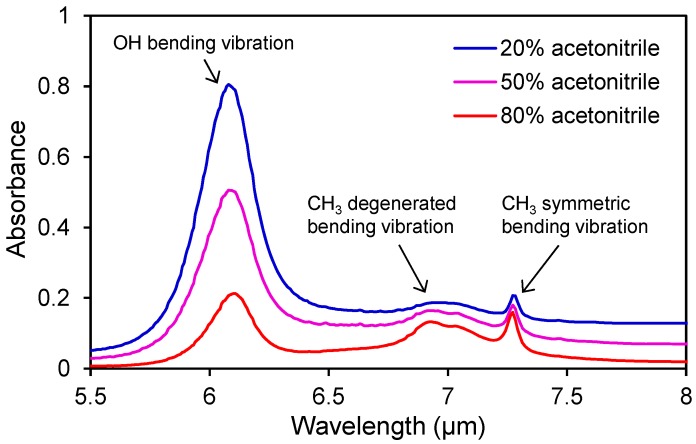
IR absorption spectra of the acetonitrile mixture used in reversed-phase liquid chromatography.

The laser wavelength must be properly selected for efficient and subsequent ion yield due to the characteristic absorption of the solvent in the mid-infrared region. Figure 3 shows the absorption spectra of a typical mobile-phase in reversed phase liquid chromatography, which includes water, acetonitrile and a small amount of formic acid. The spectral shape of the mixed solvent depends on the mixing ratio. The O–H bending vibration around 6 µm is due to water, whereas the CH_3_ symmetric and degenerate bending vibration modes around 7.3 and 6.9 µm, respectively, are due to acetonitrile. Previous reports about laser ionization without additional matrix elements depended on the strong absorption of water [20,21]. Herein, we tried to utilize a matrix containing water and acetonitrile using the mid-infrared tunable laser, which covers a wide wavelength range of 6–7 µm.

**Figure 4:**
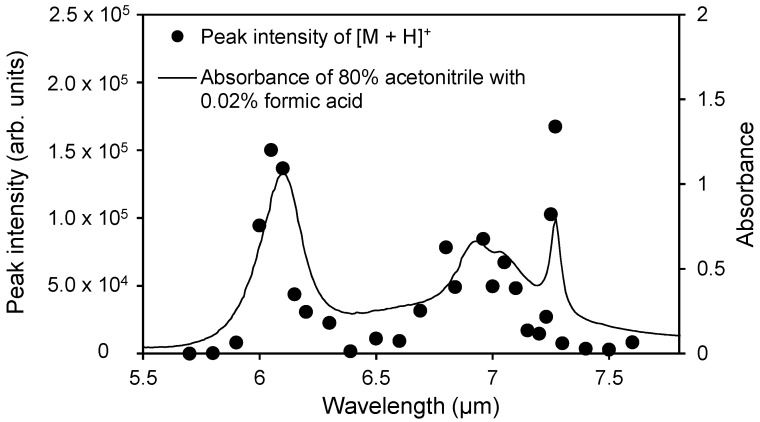
Wavelength dependence of the peak intensity of [M + H]^+^ of angiotensin II from an 80% acetonitrile aqueous solution and the IR absorption spectrum of the 80% acetonitrile aqueous solution. The electric potential of the frit and the capillary temperature were set to 2.5 kV and 270 °C, respectively.

Figure 4 shows the relationship between the ion signal intensity of [M + H]^+^ and the IR absorption spectrum. Mainly two local maxima are observed in the plot of the ion signal intensity at the respective absorption peak wavelengths of water and acetonitrile. In addition, these peak wavelengths are very similar to the case of an aqueous solution on the sample plate. These results indicate that acetonitrile can act as a matrix by using a laser with the wavelength that corresponds to the matrix absorption peak.

In general, ESI is taken as one of the most useful techniques for the production of molecular ions also in LC/MS. In the gradient analysis in which the mixing ratio of the organic solvent in an aqueous solution is elevated as the analysis proceeds, an excessive concentration of acetonitrile decreases the ionization efficiency of ESI. On the other hand, our novel method using a mid-infrared laser can utilize both water and acetonitrile as the “ionization support agent”. Therefore, the appropriate wavelength can be selected based on the eluent condition of HPLC in LC/MS with this ionization technique. Even in the case of gradient LC/MS, the center wavelength of the peaks is almost constant; even the mixing ratio is changed, as shown in Figure 3. Thus, gradient LC/MS should be possible using a fixed laser wavelength.

### 2.3. Dependence of the Ionization Efficiency on the Mixing Ratio of Water and Organic Solvent

**Figure 5:**
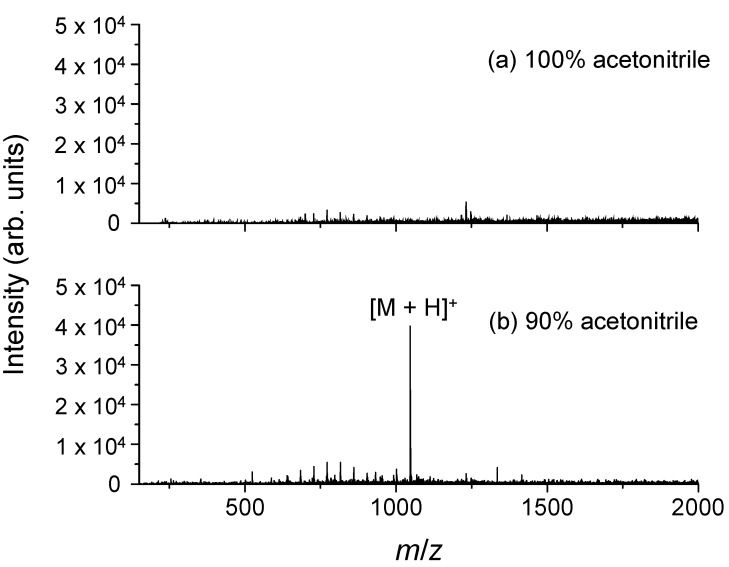
Typical mass spectra of angiotensin II dissolved in (**a**) 100% acetonitrile and (**b**) a 90% acetonitrile aqueous solution upon irradiation with a mid-IR laser at a wavelength of 7.27 µm, which corresponds to the absorption peak of the CH_3_ symmetric bending vibration mode in acetonitrile. The electric potential of the frit and the capillary temperature were set to 2.5 kV and 270 °C, respectively.

It has been previously suggested that laser absorption at 7.27 µm by acetonitrile contributes to the ionization of a dissolved peptide sample. On the other hand, the residual water solvent contained in the dried matrix crystal has been reported to influence the ionization processes in UV-MALDI [26]. Thus, we investigated whether water is necessary for the laser wavelength corresponding to the specific absorption peak of acetonitrile. Figure 5 shows typical mass spectra obtained from angiotensin II dissolved in 100% acetonitrile and a 90% acetonitrile aqueous solution. [M + H]^+^ of angiotensin II is clearly observed for the 90% acetonitrile containing 10% water, but not for 100% acetonitrile, suggesting that the presence of water is essential, regardless of whether the laser wavelength is fixed at the absorption peak of another solvent (acetonitrile in this case).

The absorption coefficients of the solvent at several wavelengths depend on the mixing ratios of the solvents. A larger mixing ratio of water leads to a stronger absorption peak in the 6-µm range, which corresponds to the O–H bending vibration mode. Water also has a broadband absorption over the 6-µm peak. The decrease in the absorption intensity at 7.27 µm, which corresponds to the CH_3_ symmetric bending vibration mode in acetonitrile, is almost countered by increasing the broadband absorption of water. Therefore, laser absorption by the solvent matrix may become constant even if the mixing ratio of the solvent (the eluent of HPLC) changes temporally according to the gradient program.

**Figure 6:**
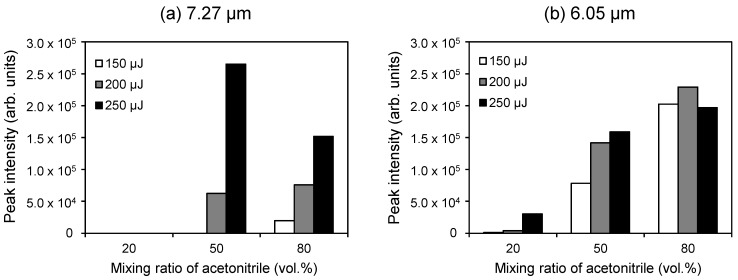
Relationships between the ion signal intensity of [M + H]^+^ of angiotensin II and the mixing ratio of acetonitrile in an aqueous solvent with laser wavelengths of (**a**) 7.27 µm and (**b**) 6.05 µm. The electric potential of the frit and the capillary temperature were set to 2.5 kV and 270 °C, respectively.

In conventional LC/MS using an ESI source, the fluctuating ionization efficiency due to the solvent mixing ratio makes quantitative analysis difficult. Hence, we investigated the dependence of the solvent mixing ratio on the ionization efficiency when using a 7.27-µm laser wavelength. Figure 6 shows the relationship between the ion signal intensity of [M + H]^+^ and three mixing ratios of water and acetonitrile. The ion signal intensities depend greatly on both the mixing ratio and the laser pulse energy.

We also investigated the influence of the solvent mixing ratio on the ion efficiency for the wavelength corresponding to water absorption (6.05 µm). Similarly, the mixing ratio affects the ion signal intensity of [M + H]^+^ (Figure 6). The 80% acetonitrile aqueous solution gives the strongest ion signal of [M + H]^+^ despite having the weakest absorption at 6.05 µm (Figure 3), indicating that the absorption intensity is not the only factor contributing to the ionization process. It is possible that the volatility and surface tension of the solvent may influence the desorption efficiency. Water has a relatively high surface tension and a lower volatility compared to other organic solvent. If the surface tension and volatility influence the ionization process, desolvation would produce ions. All mass spectrometers with an atmospheric pressure ion source have a heated capillary or a skimmer to promote desolvation from charged droplets containing sample molecules. Thus, the capillary temperature may have a considerable effect on ion production.

### 2.4. Relationship between the Generation of Multiply Charged Ions and the Desolvation Temperature

The above results suggest that the desolvation temperature and ion production are related. Thus, we investigated the relationship between the temperature of the heated capillary in an ion trap mass spectrometer (LCQ Classic, Thermo Finnigan, CA, USA) and the production of multiply-charged ions of peptides. Mass spectra were obtained from a mixture of three peptides (angiotensin II, P_14_R and ACTH (adrenocorticotropic hormone) Fragment 18–39) dissolved in an 80% acetonitrile aqueous solution with 0.01% formic acid.

In previous research using the AP-IR-MALDI of peptides and proteins [27], multiply-charged ions were produced instead of singly-charged ions, which are chiefly observed in UV-MALDI. In addition, it was recently reported that liquid AP-UV-MALDI enables stable ion yields of multiply-charged ions of peptides and proteins [28].

**Figure 7:**
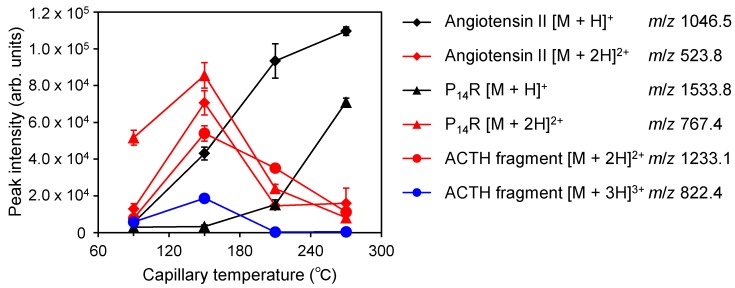
Relationship between the signal intensity of the protonated peptide ions and the temperature of the heated capillary in the mass spectrometer. The electric potential of the frit was set to 2.5 kV.

This ionization method produces multiply-charged ions, whose distribution drastically depends on the capillary temperature (Figure 7). For a capillary temperature of 270 °C, the mass spectrum contains mainly singly-protonated ions [M + H]^+^, but the intensities of doubly-charged ion [M + 2H]^2+^ and triply-charged ion [M + 3H]^3+^ increase as the temperature decreases. These results indicate that the ion production process in 6–7-µm atmospheric pressure laser desorption/ionization using a solvent matrix occurs inside the heated capillary and not at the laser irradiation point. Usually, in UV-MALDI, ions are produced in or form a dense plume containing the matrix and analytes in the gas phase. Thus, the mechanisms of the ionization process in conventional MALDI and 6–7-µm atmospheric pressure laser desorption/ionization using a solvent matrix should differ.

On the other hand, the distribution of the ion valences produced by an electrospray shifts to the high-valence side, according to the increment of the vaporization rate from charged droplets [29,30]. On the other hand, a higher vaporization rate from charged droplets causes the formation of more highly charged ions in electrospray ionization [29,30]. In ESI, multiply-charged ions are produced due to the rapid desolvation from the charged droplets and the condensation of charge. Conversely, slow desolvation may result in poorly charged ions. ESI and continuous flow AP-IR-MALDI atmospheric pressure laser desorption/ionization are completely opposite in this regard. However, both have a commonality: they need the desolvation from charged droplets, which is not required for conventional MALDI. However, the mechanism is unclear at this time, and a more detailed study is necessary.

These investigations suggested that the laser might induce not only the desorption of a charged droplet, but ionization. On the other hand, for example, simply heating is one of the choices for just desorption. However, probe heating preferentially would desorb highly volatile compounds. As a result, non-volatile compounds remain on the probe. In this regard, the use of laser desorption could provide an important benefit that every compounds in the solution would be desorbed regardless of the volatility.

## 3. Experimental Section

### 3.1. Mid-Infrared Tunable Laser Using Difference-Frequency Generation (DFG)

A mid-IR tunable laser using difference-frequency generation (DFG) was used for ionization [31]. The DFG laser was developed by Kawasaki Heavy Industries, Ltd. (Kobe, Hyogo, Japan), and RIKEN (Wako, Saitama, Japan). Laser pulses from a Nd:YAG laser with a wavelength λ_1_ of 1.064 µm and a tunable Cr:forsterite laser with a wavelength λ_2_ of 1.19–1.32 µm were synchronized and mixed in two nonlinear optical crystals (AgGaS_2_). Consequently, the mid-IR output of DFG with a wavelength λ_DFG_ = (1/λ_1_ − 1/λ_2_)^−1^ was obtained. This laser system had a tunable wavelength range of 5.50–10.00 µm, and wavelength tuning with a minimum interval of 0.01 µm was automatically controlled by a computer. The laser pulse width was about 5 ns, and the pulse repetition rate was 10 Hz. In the experiments, the laser pulse energy ranged between 250 and 400 µJ at the sample surface.

### 3.2. Ion Trap Mass Spectrometer with an Atmospheric Pressure Laser Ion Source

All experiments were conducted with an ion trap mass spectrometer (LCQ Classic, Thermo Finnigan, CA, USA) integrated with a self-assembled continuous flow atmospheric pressure laser ion source.

**Figure 8:**
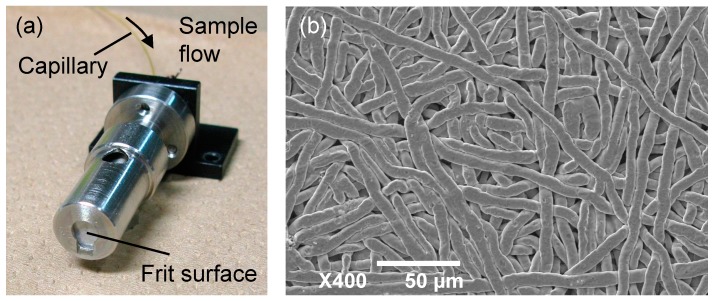
(**a**) Photograph of the frit probe and (**b**) scanning electron microscope image of the frit surface.

The sample probe was a porous stainless steel substance called a “frit” (Figure 8). A frit has been used with a conventional ionization technique using a neutral beam and some viscous matrices, called fast atom bombardment (FAB) [32,33]. These conditions realized continuous flow ionization, which was used as the interface of LC/MS [34,35,36]. In addition, a frit has applied to a modified MALDI technique using an infrared laser, named continuous flow IR-MALDI [37]. Although ionization by frit-FAB or continuous flow IR-MALDI was conducted under a high vacuum, in this work, the frit was subjected to atmospheric pressure in continuous flow laser ionization.

**Figure 9:**
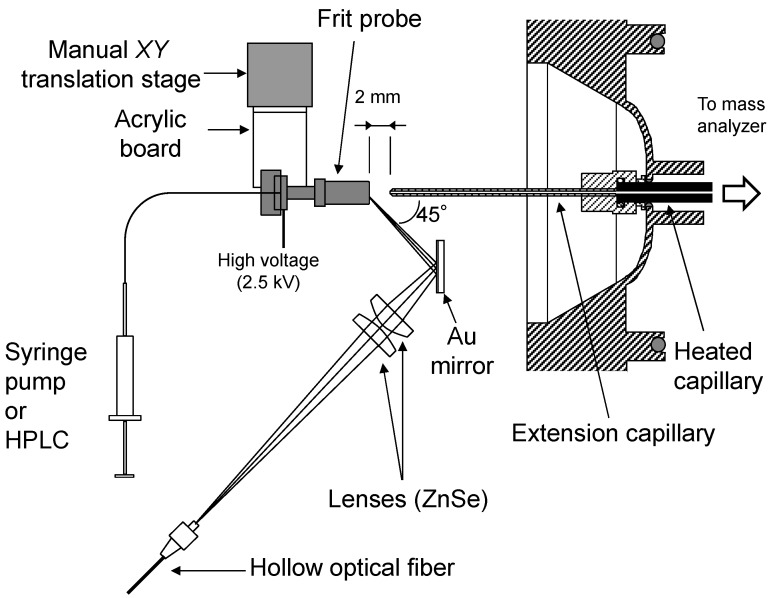
Schematic of the ion source for continuous flow atmospheric pressure laser/desorption ionization using a frit probe integrated with the ion trap mass spectrometer.

Figure 9 schematically depicts the measurement system. The output of the DFG laser was introduced into a hollow optical fiber with an inner diameter of 700 µm using an off-axis parabolic mirror to deliver the DFG laser to the ion source. The laser light from the hollow optical fiber was focused onto the sample plate using two ZnSe plano-convex lenses. The incident angle of the laser against the surface of the frit was about 45°. To calculate laser fluences on the frit surface, the knife-edge method measured the focused laser spot size, which was approximately 0.16 mm^2^.

An extension capillary was connected to the heated capillary of the mass spectrometer to bring the ion inlet close to the frit surface. The length and inner diameter of the extension capillary were 80 and 0.6 mm, respectively, and the temperature of the heated capillary was set to 270 °C. The frit probe mounted on a manual *XY* translation stage was located 2 mm from the tip of the extension capillary. It is supposed that the potential of the frit probe should have an influence on the ion production. However, the effect of the sample plate voltage on the atmospheric ionization using a 3-µm-band mid-infrared laser has already been reported elsewhere [38]. Thus, a static high voltage of 2.5 kV was applied to the frit probe by connecting the voltage source originally used for the ESI nozzle [39].

The ion injection time of the ion trap was set to 300 ms, and ions were produced by the irradiation of the DFG laser in the meantime. A raw mass spectrum was acquired by three micro-scans of the ion trap, and the mass spectra shown in Figure 1 and Figure 5 were obtained from the averaging of 50 raw mass spectra.

### 3.3. Materials and Methods

Human angiotensin II (A8846), P_14_R (synthetic) (P2613) and human ACTH Fragment 18–39 (A8346) were purchased from Sigma-Aldrich (St. Louis, MO, USA). Distilled water (049-16787) was purchased from Wako Pure Chemical Industries Ltd. (Osaka, Japan). All were used as received. These peptides were dissolved in 20%–90% acetonitrile aqueous solutions with 0.005%–0.04% formic acid (11-0780-5, Katayama Chemical Industries Co., Ltd., Osaka, Japan) at a concentration of 10 pmol/µL. Otherwise, a peptide was dissolved in 100% acetonitrile.

Sample solutions were delivered to the frit probe via a syringe with a 250-µL capacity (Unimetrics, IL, USA) and an LCQ classic syringe pump. The sample flow rate was controlled in the range of 3–20 µL/min. A sample solution was pumped at a constant flow rate during laser irradiation.

The mass spectra shown in Figure 1 and Figure 5 were averaged 50 times for only the reduction of noise. The temporal changes in the ion signal intensity were investigated by plotting the intensities in the respective spectra. The heated capillary was set at 90, 150, 210 or 270 °C.

## 4. Conclusions

The continuous flow atmospheric pressure laser ion source using a frit and a 6–7-µm-band mid-infrared tunable laser is capable of direct ionization from a continuous flow with a high temporal stability. This modified ion source requires no additional matrix and utilizes water or acetonitrile as the solvent matrix at several absorption peak wavelengths (6.05 and 7.27 µm).

In addition, the effects of the solvent mixing ratio on the ionization efficiency were investigated. The ion signal intensity depends on the mixing ratio at wavelengths of 6.05 and 7.27 µm. In the case of 6.05 µm, the ion signal intensity obtained from the 20% acetonitrile aqueous solution is lower than that from the 50% and 80% acetonitrile aqueous solutions despite their relatively high absorbance at 6.05 µm, indicating that not only the intensity of laser absorption, but also the volatility and surface tension may affect the desorption efficiency.

The distribution of multiply-charged peptide ions is extremely sensitive to the temperature of the heated capillary, which is the inlet of the mass spectrometer. This suggests that ions are produced from the charged droplet after desorption of the solvent matrices (water or acetonitrile). The solvent matrices used in this work were broadly used as mobile-phase in reversed-phase liquid chromatography. Thus, this ionization method has the potential for the interface of LC/MS as an alternative for ESI.
